# Shared data for intensity modulated radiation therapy (IMRT) optimization research: the CORT dataset

**DOI:** 10.1186/2047-217X-3-37

**Published:** 2014-12-12

**Authors:** David Craft, Mark Bangert, Troy Long, Dávid Papp, Jan Unkelbach

**Affiliations:** Massachusetts General Hospital, Harvard Medical School, 02114 Boston, MA USA; German Cancer Research Center (DKFZ), 69120 Heidelberg, Germany; University of Michigan, 48109 Ann Arbor, Michigan USA

**Keywords:** IMRT, Optimization, Radiation therapy, Beam angle optimization, VMAT, Treatment plan optimization

## Abstract

**Background:**

We provide common datasets (which we call the CORT dataset: common optimization for radiation therapy) that researchers can use when developing and contrasting radiation treatment planning optimization algorithms. The datasets allow researchers to make one-to-one comparisons of algorithms in order to solve various instances of the radiation therapy treatment planning problem in intensity modulated radiation therapy (IMRT), including beam angle optimization, volumetric modulated arc therapy and direct aperture optimization.

**Results:**

We provide datasets for a prostate case, a liver case, a head and neck case, and a standard IMRT phantom. We provide the dose-influence matrix from a variety of beam/couch angle pairs for each dataset. The dose-influence matrix is the main entity needed to perform optimizations: it contains the dose to each patient voxel from each pencil beam. In addition, the original Digital Imaging and Communications in Medicine (DICOM) computed tomography (CT) scan, as well as the DICOM structure file, are provided for each case.

**Conclusions:**

Here we present an open dataset – the first of its kind – to the radiation oncology community, which will allow researchers to compare methods for optimizing radiation dose delivery.

## Background

The goal of radiation therapy for cancer treatment is to irradiate the tumorous regions of the body with sufficiently high levels of radiation while sparing nearby healthy tissues as much as possible. In the mid 1990s a technique known as intensity modulated radiation therapy (IMRT) emerged which further enables tailoring of the 3D dose distribution inside the patient. Along with this extra freedom comes the need for mathematical optimization, and over the last 20 years a large amount of research has produced over of 600 papers (a conservative estimate based on a PubMed search for the words “IMRT” and “optimization” in the title or abstract) revolving around this topic.

A deficiency in the field has been the lack of common datasets for researchers to test their algorithms on. As such, most new algorithm papers simply state the algorithm and demonstrate it, but the reader is left to wonder how this algorithm compares with other approaches to the same problem. Furthermore, the raw data that was used for a specific study is never provided as part of the publication, for reasons such as data size, involvement of commercial software products, and protection of data privacy for individual patients.

With this paper, we want to address these issues and provide the basis for meaningful benchmarking of IMRT optimization algorithms. Specifically, our initiative aims at resolving the following shortcomings:

- Patient cases used in different papers differ greatly in the geometry of their targets and critical structures. A technique that works on an “easy” patient may not work as well on a “challenging” patient and vice versa.
- Research papers make different assumptions when deriving plan optimization data from the patient’s planning computed tomography (CT) scan. This includes the dose calculation method, spatial resolution of the dose and beamlet grid, planning goals, delivery modality, *etc*. These data are generated in-house and are not shared with the research community.
- New researchers in the field may not have access to clinical patient datasets.

**Figure 1:**
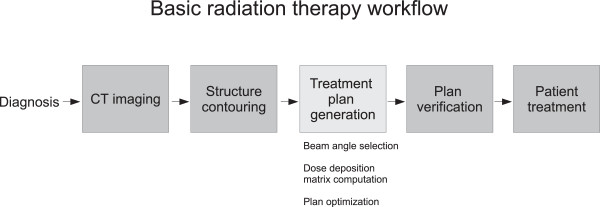
**Radiation therapy workflow.** CT imaging provides the 3D image set of the patient. This image set is used by the physician to draw the contours of the tumor and the nearby important healthy organs. At this point this combined dataset (CT and contours) is handed off to a treatment planner who selects beam angles and proceeds with the optimization of a treatment plan. This is the step that we model and provide data for in this paper. In the actual clinical workflow, fluence levels that are the result of the optimization need to be converted to multi-leaf collimator positions and monitor units to form a deliverable treatment plan. At this point the deliverable treatment plan is verified, and once it passes this quality assurance step, it is used to treat the patient.

The datasets we present herein, which we call the CORT dataset (common optimization for radiation therapy), are applicable to IMRT [1–3] and its variants, including beam angle optimization (BAO) [4–7], volumetric modulated arc therapy (VMAT) [8–13], 3D-conformal optimization [14, 15], stereotactic body radiation therapy (SBRT) [16], and direct-aperture optimization (DAO) [17–22]. We refer readers to the citations for explanations of each of these (overlapping) modalities. The overall workflow of radiation therapy treatment is shown in Figure 1.

## Data description

Here, we present datasets comprising three anonymized cancer patient cases and one standard IMRT phantom. For each of the four cases, we include the original DICOM CT image as well as the DICOM RTStruct file containing the contours of targets and organs at risk. These files are made available for viewing results, although they are not necessary for optimization. In addition, the DICOM files give researchers the opportunity to replan these patients in a commercial treatment planning system. All further data is derived from the DICOM data.

### Voxel grid

For dose calculation, the original CT image is downsampled to a lower resolution. The final resolution and size of the dose grid in three dimensions is stored in a text file named CTVOXEL_INFO.txt. Each voxel in the 3D dose grid is assigned a voxel index, which is used in optimization data described below. The coordinate system and the conversion of voxel indices to spatial location is described in Methods. For standard optimizations, voxel positions are not needed. However, they are required for visualization of the dose distribution, and are useful for implementing objective functions which require spatial information, for example a dose penalty which depends on the distance from a normal tissue voxel to the patient’s tumor. This file also contains the isocenter location. The isocenter denotes the point in space about which couch and gantry rotate to achieve different beam orientations.

### Beamlet grid

The incident fluence is discretized into a rectangular grid of beamlets. We use a beamlet size of 1cm × 1cm for all cases except for the head and neck case, for which we use 0.5 cm × 0.5 cm. The isocenter is identical for all beam directions and is located in the center of mass of the union of all target volumes. The set of beamlets for which dose is calculated is based on an isotropic 2.5 mm expansion of the union of all targets. A beamlet is included in the fluence map if its central axis intesects the enlarged target. In a post-processing step, we ensure that the beamlet grid is consecutive. If beamlets are missing from the fluence map, causing a hole across a multi-leaf collimator (MLC) row, these beamlets are added and their dose distribution is calculated. This issue arises for example in the head and neck case with disconnected targets on either side of the neck. Missing beamlets could be problematic for sliding window IMRT and VMAT optimization approaches, where the MLC leaves would potentially slide over those beamlets. The beamlet grid coordinate system is described in Methods.

### Optimization data

All binary formatted data are saved from Matlab as *.mat files (we have used Matlab version 7.14.0.739, R2012a)^a^. In this way, data can either be read into Matlab, Octave or Python using the scipy package. Instructions for reading in the data are given in the Methods section. For treatment plan optimization, we provide the following files for each patient.

#### Voxel lists

The voxel list files contain the indices of the voxels which are inside each geometrically contoured structure. The information is stored as a list of integers in the files {structure name}_VOILIST.mat. The format thus allows for overlapping structures, in other words a given voxel index can be contained in multiple voxel lists.

#### Beamlet information

**Figure 2:**
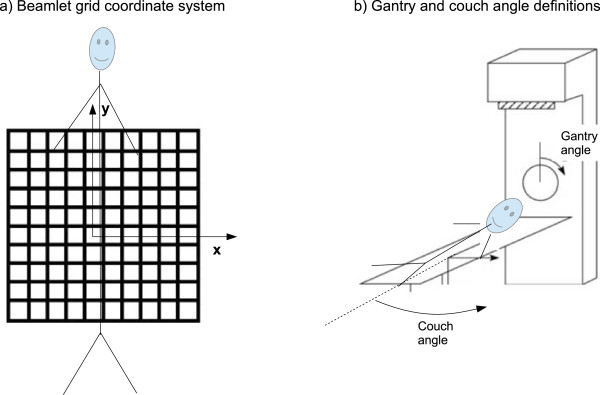
**Beamlet grid, gantry angle, and couch angle definitions.** A picture displaying **(a)** the beamlet coordinate system used, for a sample beam placed at gantry angle 0 and couch angle 0, and **(b)** the definitions of the gantry and the couch angle.

For each (gantry angle, couch angle) pair, a beam information file with the file name Gantry{gantry angle}_Couch {couch angle}_BEAMINFO.mat is provided. The file contains the following information:

- couch angle
- gantry angle
- number of beamlets
- number of non-zeros in the dose-influence matrix (see the next section; this value is helpful for pre-allocating memory to store these matrices)
- A vector of the *x* position of each of the beamlets (using the gantry head coordinate system, see Figure 2).

- A vector of the *y* position of each of the beamlets (see Figure 2).

Geometric beamlet information is not necessary for the most primitive type of IMRT optimization, but when a fluence map smoothing term is to be included, for example, see [23, 24] or for VMAT and DAO (where “apertures” are created by combining adjacent beamlets), it is necessary to know the geometric location (*x*, *y*) of each of the beamlets. See also Figure 2(a). Although a non-zero collimator angle can be useful for VMAT delivery and standard IMRT where delivery time is of high concern, for simplicity we have only used a collimator angle of 0 for these datasets and so do not include collimator angle as a field in the BEAMINFO files.

#### Dose-influence matrix

The dose influence matrix *D*_*ij*_ is the main entity used for optimization. It contains the dose delivered to each voxel *i* per unit intensity of beamlet *j*. We provide the dose influence matrix in units of Gray per monitor unit (Gy/MU)^b^. The dose-influence matrix is stored in separate files for each (gantry angle, couch angle) pair in files named Gantry{gantry angle}_Couch{couch angle}_D.mat. The beamlet order (index) is as they are ordered in the (*x*, *y*) data in the corresponding BEAMINFO file. Each of the dose-influence files contains a single matrix called *D*, which is a Matlab sparse matrix ^c^. We use CERR version 4.4 (Computational Environment for Radiotherapy Research) [25] to produce the dose influence matrices for each case. CERR uses a pencil beam type dose calculation algorithm referred to as the quadrant infinite beam (QIB) model [26, 27]. This method uses pretabulated integration values to allow for a fast computation of *D*_*ij*_. We use the default values in the CERR IMRT GUI regarding the specifics of the dose computation (Gaussian primary and scatter radiation, exponential scatter method, 6 Megaelectron-volts beams).

The dose to voxel *i* is given by
1

where *x*_*j*_ is the fluence value of the *j*th beamlet.

### Hints for CERR users

To generate the optimization data, the DICOM CT data was imported into CERR and the CT scan was then resampled to the voxel sizes shown in Table 1. This was done using the CERR command downSampleScan. Once the data was downsampled, the CERR IMRTP module was used to create the dose-influence matrices. Our group has modified this code to allow for couch rotations. The dose-influence matrix was then extracted from the internal CERR data structure and rescaled to units of Gy/MU. We also provide a Matlab.mat which is generated by CERR when saving the patient. This file contains, among other attributes, the downsampled CT scan, which has the same resolution as the dose grid, and can be used for visualization. For size purposes, this file does not contain the *D*_*ij*_ matrices.

**Table 1:** **Summary of patient characteristics**

	TG119	Prostate	Liver	Head and neck
Number of beam angles	5	180	56	1983
Total number of beamlets	418	25,404	3678	2,257,507
Noncoplanar	no	no	yes	yes
Beamlet size [cm]	1 ×1	1 ×1	1 ×1	0.5 ×0.5
Voxel resolution (LR,AP,SI) [mm]	(3.0, 3.0, 2.5)	(3.0, 3.0, 3.0)	(3.0, 3.0, 2.5)	(3.0, 3.0, 5.0)
Voxel grid size (LR,AP,SI)	(167,167,129)	(184,184,90)	(217, 217,168)	(160,160,67)
Number of target voxels	7429	9491	6954	25,388
Number of voxels in patient	599,440	690,373	1,927,357	251,893
dataset size	25 MB	1.9 GB	560 MB	64 GB

Number of target voxels for the head and neck case is for the union of the three planning target volume (PTV) structures. AP = anterior-posterior, LR = left-right, SI = superior-inferior.

### The four cases

**Figure 3:**
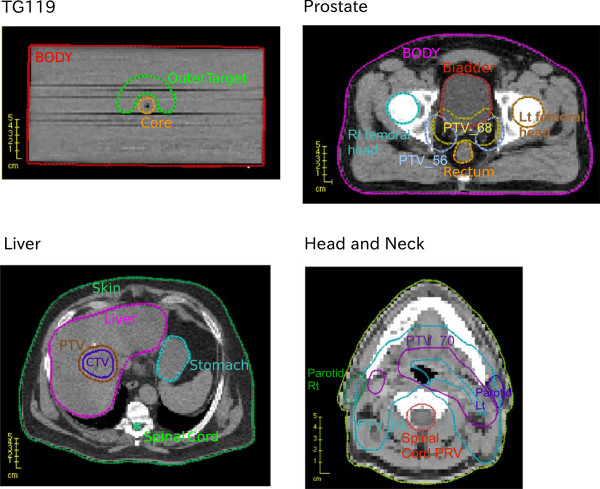
**Axial views of the four cases.** CT and structures for the four cases for a representative CT slice (which shows some but not all of the structures included in the dataset).

We provide data sets for four patients of different sizes to support a variety of radiotherapy planning problems and represent typical treatment sites. The main characteristics of all datasets are summarized in Table 1. A representative transversal slice through the CT, illustrating the geometry of target and organs at risk (OARs) for each case, is shown in Figure 3.

#### TG119 dataset

The first case we use is a phantom provided by the American Association of Physicists in Medicine Task Group 119 for use in institutional IMRT commissioning (i.e. readying a clinic for IMRT treatments) [28]. This phantom has several sets of contours for various IMRT treatment planning tests, but we only use three of the contours: a C-shaped target (called “OuterTarget”), an OAR that the target wraps around (“Core”), and the external contour of the phantom itself (“BODY”).

For this case we provide five equispaced coplanar beams (coplanar refers to beams where the couch angle is fixed at 0°) at gantry angles 0°, 72°, 144°, 216°, and 288°. This serves as our small dataset. The total number of beamlets at each respective angle is 98, 70, 90, 90 and 70, for a total of 418 beamlets.

#### Prostate

The prostate case serves as one of our two medium size datasets. We generate 180 equispaced coplanar beams, thus this data set serves as a test case for VMAT algorithms. Using a beamlet resolution of 1 cm × 1 cm, the total number of beamlets is 25,404. There are two targets for the prostate case. The highest prescription dose target, PTV_68, is a geometric expansion of the prostate. The lower dose target, PTV_56, is an expansion around the prostate and the lymph nodes.

#### SBRT liver case

This is the first non-coplanar case we present. We originally generate 162 (gantry, couch) angle pairs such that the entry angles are evenly scattered over a sphere corresponding to an average angular spacing of 16°. This was done using a Matlab routine called GridSphere available from the File Exchange portion of the MathWorks website. We then eliminate beams that have either entrance or exit doses through the first slice of the CT since if this is the case, the full dose deposit of the beam is not properly accounted for. This leaves 56 beams in the dataset, with a total of 3678 beamlets. Note that given a particular linac, some gantry/couch angle combinations may not be allowed due to mechanical collisions. Since this is linac specific, we have not attempted to eliminate such beams, and instead leave it to the reader to keep this in mind if modeling an actual clinical delivery situation.

#### Head and neck

**Figure 4:**
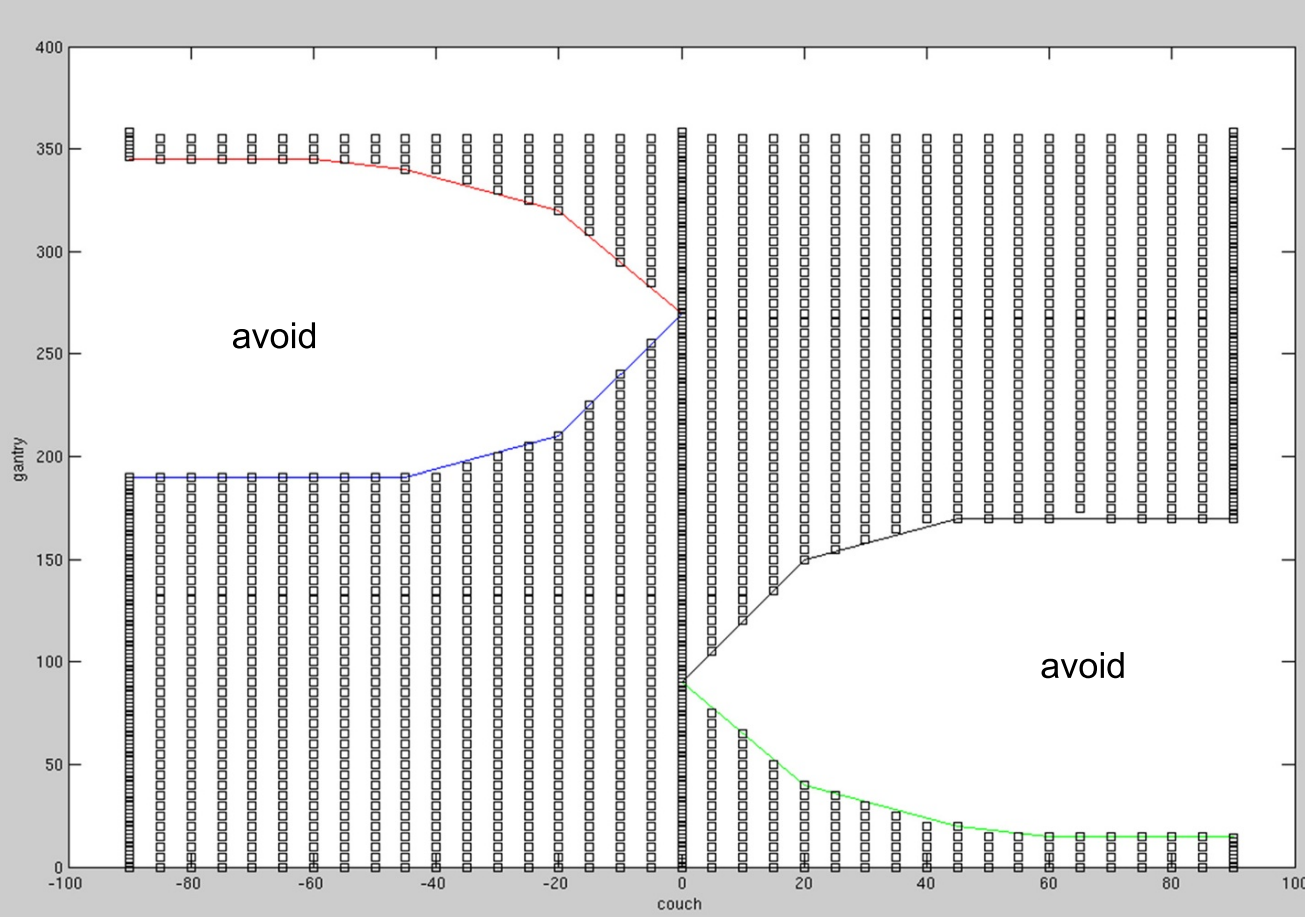
**Elimination map for head and neck angles.** A picture displaying the couch/gantry angle pairs that were eliminated due to the beam entering the inferior CT slice, thus causing an incorrect dose computation.

This serves as our large dataset. The CT and structures are obtained from the publicly available research set [29]. This set was created with non-coplanar VMAT in mind and creates a full set of equispaced beams for a variety of couch angles. The couch angles are -90° to 90° in increments of 5°. At the couch angles -90, 0, and 90, we place beams at a 2° gantry spacing. At the other couch angles we use a 5° gantry spacing. A 2° resolution is the clinical standard gantry discretization for computing VMAT doses; 5° is adequate for research purposes. We eliminate beams that enter through the inferior-most CT slice, where the CT scan ends. The elimination map is shown in Figure 4. This leaves 1983 beam angles used, with a total of 2,257,507 beamlets. The beamlets for this case are 0.5 cm × 0.5 cm. The voxel resolution is 3 mm × 3 mm × 5 mm. Unfortunately we cannot provide data at a higher resolution in the sup-inf direction due the sparse resolution of 5 mm in the original CT. Nonetheless, this is perfectly adequate for computation research purposes.

## Analyses

As a data verification step, we give dose distribution statistics for the ones solutions (*x*_*j*_=1 for all *j*) for all cases. We give the dose statistics for two volumes from each case, one target and one critical structure, see Table 2. In the Methods section we provide the Matlab code used to perform this calculation.

**Table 2:** **Dose statistics for all cases, for two selected structures, for the ones solution (all beams), i.e.**

Case	Structure name	Minimum dose	Mean dose	Maximum dose
TG119	Core	0.026798	0.050724	0.053313
	OuterTarget	0.049379	0.051067	0.052702
Prostate	PTV_56	1.3015	1.3631	1.4089
	Bladder	0.66549	1.2753	1.3863
Liver	Heart	0.0003963	0.093117	0.4388
	PTV	0.37532	0.41629	0.48094
Head and Neck	PTV_70	19.5394	21.0998	23.1338
	PAROTID_LT	8.7997	20.3354	23.7127

This serves as a data consistency check for users. All doses in Gy.

### Optimization demonstration and results

#### Description of the IMRT optimization problem

Here we describe what is known as the fluence-based IMRT optimization problem. This an idealized version of the actual IMRT treatment planning problem, but is commonly used to develop algorithms and indeed is possible to use in clinical settings (e.g., [30]).

For a set of beams, we assume the *D* matrix represents the entire set of beamlets from all the beams. That is, below *D* is interpreted as a concatenation of the individual *D* matrices from each beam. This notationally simplifies the problem, allowing us to avoid looping over the beams, instead we just loop over all beamlets. Let *d* be the vector of voxel doses, and let *x* be the vector of beamlet fluences. The key mapping is the linear relationship between the beamlet vector and the dose distribution given in Equation (1). Writing this dose calculation in the form of a matrix-vector product *Dx*=*d*, a generic formulation of the IMRT optimization problem is as follows:
2

A specific example that would give rise to a linear program would be to choose as *f*(*d*) the mean dose to a critical structure, and to invoke upper bounds for all voxels and additional lower bounds for the target voxels via the constraint set *C*. A typical quadratic formulation would set goals for every voxel (e.g., prescription dose to all target voxels and 0 to all other voxels) and minimize the squared deviation from those levels.

BAO, DAO and VMAT formulations put additional restrictions on the *x* vector. For example for BAO, one might restrict that a total of five beams are used at most, and thus integer variables could be added to this formulation to control the maximum number of active beams/beamlets.

#### Examples of linear programming formulations

In Tables 3, 4, 5 and 6, we present simple linear programming formulations and summary results for each of the four cases. These formulations are not meant to produce quality treatment plans but are rather selected to be simple to implement and thus reproduce results as a baseline.

**Table 3:** **Linear programming formulation and solution statistics for the TG119 case, all five beams used**

Objective	min (mean Core + mean BODY)
Constraints	OuterTarget >= 1
	OuterTarget <= 1.2
	Core <= 1.2
Results	mean Core = 0.2489
	mean BODY = 0.1021

All doses in Gy.

**Table 4:** **Linear programming formulation and solution statistics for the Prostate case, using the five beams at gantry angles 0°, 72°, 144°, 216°, and 288°**

Objective	min (mean Rectum + 0.6*mean Bladder+ 0.6*mean BODY)
Constraints	PTV_68 >= 1
	x <= 50
Results	mean Rectum = 0.2842
	mean Bladder = 0.4035
	mean BODY = 0.0905

All doses in Gy.

**Table 5:** **Linear programming formulation and solution statistics for the Liver case, using the seven beams at (gantry, couch) angles (58°, 0°), (106°, 0°), (212°, 0°), (328°, 0°), (216°, 32°), (226°, -13°), (296°, 17°)**

Objective	min (mean Liver + mean Heart+ 0.6*mean entrance)
Constraints	PTV >= 1
	x <= 25
Results	mean Liver = 0.1771
	mean Heart = 0.1258
	mean Entrance = 0.0186

All doses in Gy.

**Table 6:** **Linear programming formulation and solution statistics for the head and neck case, using five gantry angles at couch = 0° (0°, 72°, 144°, 216°, and 288°) as well as five gantry angles at couch = 20° (180°, 220°, 260°, 300°, 340°)**

Objective	min (mean Left Parotid + mean Right Parotid)
Constraints	All PTVs >= 1
	spinal cord <= 0.5
	brainstem <= 0.5
	x <= 25
Results	mean Left Parotid = 0.4959
	mean Right Parotid = 0.3437

All doses in Gy.

## Discussion

We provide four datasets for radiotherapy treatment plan optimization. The datasets are meant to serve several purposes:

- We provide datasets for researchers in the optimization community who may not have access to patient data.
- Advanced problems like BAO, DAO and VMAT represent non-convex or combinatorial problems which typically cannot be solved to optimality. Thus, solution approaches are heuristics, and different methods can only be compared meaningfully based on common datasets, where differences due to patient geometry and dose calculation are eliminated.
- The datasets can serve as benchmark cases for the development of fast and efficient solvers customized to fluence map optimization and its variants. This development may also benefit other radiotherapy planning problems such as robust optimization in proton therapy and adaptive re-planning in online image guided radiotherapy. Our datasets do not per se support these specific problems. However, such applications rely on very fast optimization methods that can handle large instances of optimization problems of the form (2).

### Solution reporting

We recommend that researchers share results in the maximally transparent and reproducible manner. This includes the statement of the full optimization problem that was solved. In addition, the solution should be shared in the form of fluence maps, from which the dose distribution and all dose measures can be derived. The details of the solution reporting may depend on the application:

- For IMRT fluence map optimization, the solution is the vector of beamlet intensities *x* at each beam (gantry/couch pair) that is used in the solution. As such, we recommend the following file format for users to report and share solutions. The file name should match the name of the Dij file (replacing the “_D.mat” with “_beamletSol.mat”), and should consist of fluence values stored as a vector called beamx. The beamlet solution files for the linear programs solved above are included in the data download.
- For DAO applications, the solution can be reported through an effective fluence map for each individual aperture, using the same format. Similarly, VMAT algorithms that represent extensions of DAO algorithms can report the solution in the form of effective fluence maps for all control points.

### Fluence map optimization

We have presented results for the ones solutions and for simple linear programs for the purpose of data testing and consistency. We have not included solution times since the purpose of this paper is not to present methods for fast/quality solutions to the IMRT problem, but rather to provide a set of data for the community to do such things. We used the Matlab linear programming solver (linprog) to solve the TG119, the prostate, and the liver case, but switched to CPLEX’s Matlab interface (cplexlp) to solve the head and neck case, due to its size. All cases finished in under two minutes, except for the head and neck case which took about 8 minutes.

The optimization formulation given in formulation (2) involves the linear mapping from the fluence values *x* to the voxel doses *d* as given in Equation (1). As such, provided the function *f*(*d*) is convex and the constraint set *C* is convex, the problem is a convex optimization problem. Hardware considerations, such as determining MLC positions to directly form the desired fluence maps (DAO), make the problem non-convex, as do dose-volume constraints which specify for example that only a certain number of voxels of a structure can exceed a certain dose level. The discrete form of the beam angle optimization problem, where candidate beams are pre-selected and the optimization problem is to find a subset of the beams (for example, the seven best beams) and their beamlet fluences to optimize a given objective, is a combinatorial problem, and thus is also non-convex.

### DAO and VMAT applications

In modern clinical treatment planning systems, fluence based optimization is done (at most) as an initial step. Final plan optimization involves determining aperture shapes (specified by the positions of MLC leaves) and weights. To that end, many modern planning systems apply DAO methods.

Once a segment shape is computed, the dose is linear in the segment weight. To a first approximation, the dose contribution from a segment is the sum of the contributions from the individual beamlets that constitute that segment (i.e., the information stored in the *D*_*ij*_ matrix). But better accuracy is obtained by doing a dose computation for each individual aperture shape, which involves scatter terms that can only be computed once the aperture shape is known. Using the datasets provided herein, dose calculation for an aperture is limited to approximations based on the *D*_*ij*_ matrix. Despite this limitation, this dataset can be used for DAO algorithm design. Indeed, most DAO algorithms heavily utilize the *D*_*ij*_ matrix concept for generating promising apertures [22] or for approximating gradients with respect to MLC leaf positions [20, 31]. Only a more accurate final or intermittent recalculation of an aperture’s dose distribution cannot be performed using this dataset.

Similarly, VMAT treatments need to consider MLC leaf positions in order to emulate a clinical VMAT optimizer. VMAT solvers typically strive to find a solution where the beam rotates completely around the patient on the order of minutes. As such, complete fluence modulation cannot be achieved at every angle, and MLC leaf positions must be tracked to make sure neighboring apertures are similar so that the gantry does not need to slow down excessively to move the leaves far across the treatment field. Because this dataset involves beamlet position information and couch and gantry positions, it can be used for VMAT optimization research. To include delivery time in VMAT planning optimization one must specify a dose rate. A typical value is 600 MU/min.

## Conclusion

We provide the first open dataset to the radiation oncology community, thus allowing researchers to compare methods for optimizing radiation dose delivery. The dataset comprises four patient cases from different sites. Besides CT data and structure sets, we also include dose calculation data in order to enable a one-to-one comparison of novel and existing optimization strategies for intensity modulated radiation therapy, beam angle optimization, direct aperture optimization, and volumetric-modulated arc therapy.

## Methods

**Figure 5:**
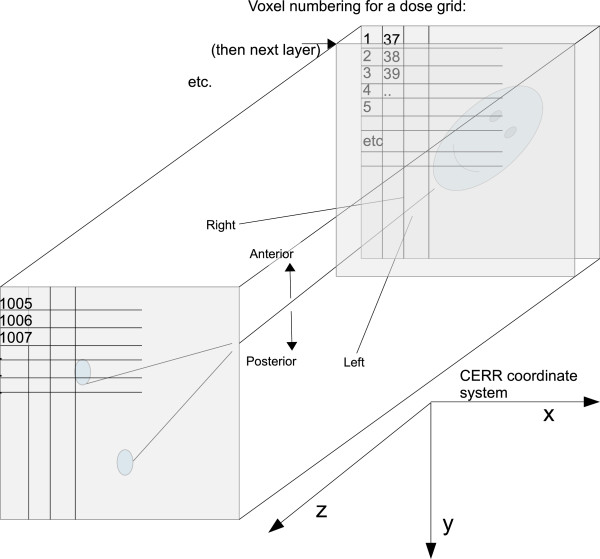
**Voxel numbering.** A picture describing the voxel numbering pattern, the patient orientation, and the CERR coordinate system.

Figure 5 shows the conversion from voxel indices to voxel locations inside the patient. All patients in the data set are in standard orientation, i.e. supine and head first. The voxel with index “1” is located most anterior, superior, and to the patient’s right. For voxel indexing, the anterior-posterior direction corresponds to the fastest changing index; the superior-inferior direction corresponds to the slowest changing index.

Figure 2(a) shows the definition of the beamlet grid. Throughout the dataset we assume a collimator angle of 0. For a couch angle of 0, the y-axis of the beamlet grid corresponds to the superior-inferior (z) direction of the patient. For a gantry angle of 0, the x-axis of the beamlet grid corresponds to the left-right direction of the patient. The beamlet grid is positioned such that the ray perpendicular to it passing through (0,0) passes through the patient iso-center. Figure 2(b) shows the definition of the positive gantry and couch angles.

### Demonstration code

For reading data into Python, the scipy package is used, and then the Matlab.mat files can be read directly. For example:


Next we include two sample code snippets to assist people in getting started with the datasets in the Matlab environment. First we give a code which computes the mean dose to a structure for the ones solution of all of the beams in the current working directory.


Next we give the code for obtaining a simple linear programming solution for the liver case. The first section of the matlab code reads the dose-influence matrix for seven selected beam angles and constructs the concatenated *D*_*ij*_ matrix. Subsequently, the voxel lists for five of the structures are imported.


The next code section constructs a linear optimization problem as described in the Analyses section. A weighted sum of the mean doses to the liver, the heart, and the normal tissue in the entrance region is minimized, subject to the constraints that every PTV voxel receives a dose larger than one. The linear program is solved using Matlab’s build-in solver linprog. The optimal fluence map is returned into the vector x.


Next, the solution to the fluence map optimization problem is saved in the recommended format:


Finally, the mean doses are reported and the 3D-dose distribution is visualized. The vector of dose values is obtained by multiplying the dose influence matrix with the beamlet intensity vector. The dose vector is then converted into a 3D-dose distribution based on the voxel numbering pattern described in Figure 5. The voxel lists for the structures are converted to 3D binary masks and plotted as contours on top of the colorwash dose display. CERR users can use the CERR function showIMDose.


## Availability of supporting data

The data supporting this article are available in the GigaScience repository, GigaDB, [32].

## Endnotes

^a^ Note that, if one were to use a more recent version of Matlab to save data for reading into Python etc, one should use the Matlab toggle -v7 during the save command.

^b^ The unit of beamlet intensity (MU) is defined such that 100 MU yields a dose of 1 Gy in 10 cm depth in water in the center of a 10 cm × 10 cm radiation field. We choose the units of Gy/MU for the dose-influence matrix in order to facilitate studies where treatment delivery time and/or variable dose rates are of interest.

^c^ The dose influence matrix can by read directly into Matlab, Octave and Python as a sparse matrix (see the Methods section). Note however that Python is 0-based whereas Matlab and Octave are 1-based. The voxels indices stored in the {structure name}_VOILIST.mat files are 1-based, i.e., the lowest voxel index is 1, as depicted in Figure 5. Thus the user has to perform the appropriate shift when using Python.

## Abbreviations

AP: Anterior-posterior
BAO: Beam angle optimization
CERR: Computational environment for radiotherapy research
CORT: Common optimization for radiation therapy
CT: Computed tomography
DAO: Direct aperture optimization
DICOM: Digital imaging and communications in medicine
Gy: Gray
IMRT: Intensity modulated radiotherapy
LR: Left-right
MLC: Multi-leaf collimator
MU: Monitor unit
OAR: Organ-at-risk
PTV: Planning target volume
QIB: Quadrant infinite beam
SBRT: Stereotactic body radiation therapy
SI: Superior-inferior
VMAT: Volumetric modulated arc therapy.
